# Editorial: The effect of heterogeneity of the network of alveolar wall tissue on airflow, interstitial flow and lung biology

**DOI:** 10.3389/fnetp.2023.1272172

**Published:** 2023-08-22

**Authors:** Akira Tsuda, Frank S. Henry

**Affiliations:** ^1^ Tsuda Lung Research, Shrewsbury, MA, United States; ^2^ Department of Mechanical Engineering, Manhattan College, Riverdale, NY, United States

**Keywords:** alveolar wall, airflow, interstitial flow, lung biology, editorial

Over inhalation, oxygen-rich air is drawn into the alveolar cavity by the expansion of the alveolar volume. The volume expansion results in an increase in the alveolar surface area. Because septal tissue is essentially incompressible, stretching of the alveolar surface area results in a thinning of the alveolar wall thickness. The reverse process happens over exhalation; that is, the surface area decreases and the wall thickness increases. The cyclic motion of the alveolar walls plays an important role in influencing the motion of fluid in the interstitial space (i.e., the space between the alveolar epithelium and vascular endothelium).

**FIGURE 1:**
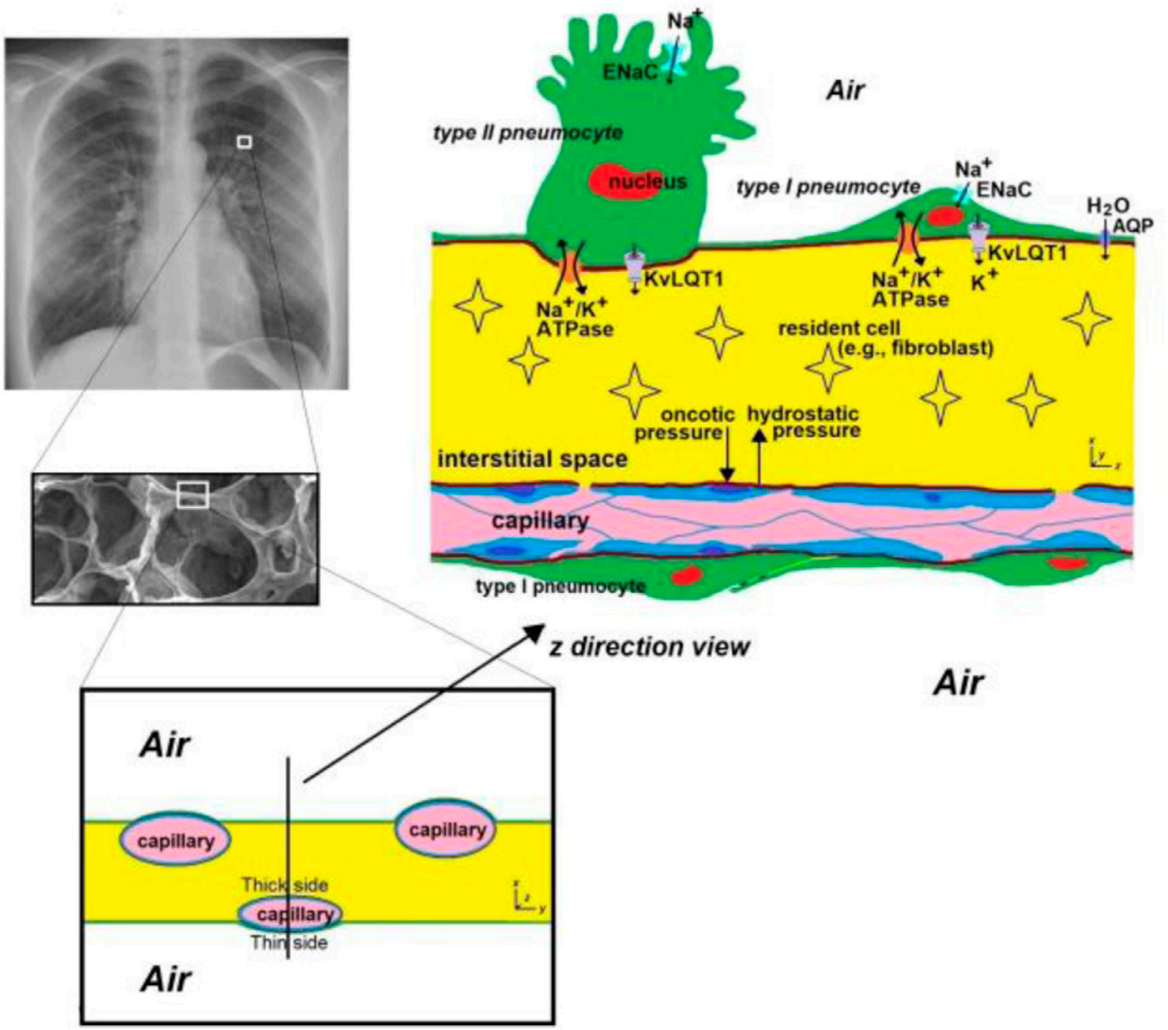
A schematic of the alveolar septa. The alveolar septa is 12 ± 3 μm thick (Gehr et al., 1978; Vasilescu et al., 2020) and is highly spatially and temporally heterogeneous. Since the capillary is not in the middle of the septal space, two types of structure exist. Where the capillary is very close to the alveolar space (noted as the “thin” side), epithelial cells and endothelial cells share a common basal lamina, resulting in the distance through which respiratory gases (O_2_, CO_2_) diffuse being <0.5 μm (West, 2012), which enhances gas exchange. On the other side of the capillary, the distance between the capillary and the alveolar space is relatively large (noted as the “thick” side), and this side contains extracellular matrix which structurally stabilizes the septa and contributes to the mechanical properties of alveolar walls. ENaC = Epithelial sodium channels. AQP = aquaporins. KvLQT1 = K+ channels. Chest radiograph (left upper) from https://en.wikipedia.org/wiki/Chest_radiograph.

The capillary network surrounding the alveoli is extensive but it does not provide a continuous, uniform, layer. Hence, the thickness and mechanical properties of the alveolar walls are not uniform. On the thin side (Figure 1), the epithelium and endothelium share one common basal lamina. This structural arrangement maximizes gas diffusion, and helps prevent fluid accumulation. On the thick side (Figure 1), extracellular matrix structurally stabilizes the septa, contributing to the mechanical properties of the alveolar walls. Dickie et al. (2007), Dickie et al. (2009) and Tsuda et al. (2019) showed that the structure of the alveolar wall changes over time. Specifically, they found that the alveolar barrier of developing lungs is more easily compromised and susceptible to foreign material influx than that of adult lungs.

Interstitial fluid delivers nutrients and oxygen to cells and transports organic wastes, damaged cells, and foreign invaders (nano particles, bacteria, viruses, *etc.*) from the interstitial space (Choi et al., 2010). Fluid enters the interstitium from the capillaries at the arterial end of the capillary bed and leaves at the venous end. The pressure gradient driving this flow varies along the interstitium, and is a combination of hydrostatic and plasma oncotic pressure between the capillaries and the interstitium. Albumin is responsible for the majority the plasma oncotic pressure (Waddell, 2009). The variation of flow along the interstitium provides another element to the heterogeneity in the alveolar wall.

Another source of heterogeneity in the alveolar wall is that the alveolar epithelium is composed of flat and thin Type I pneumocytes, and cuboidal Type II pneumocytes (Figure 1). The former covers most of the alveolar surface and is ideal for gas exchange and the latter plays a crucial role in producing and secreting pulmonary surfactant, which stabilizes the lung structure by reducing surface tension within the alveoli. The heterogeneity of the wall composition leads to non-uniform wall movement. Since any alteration of space morphology and mechanical properties influences the interstitial fluid mechanics (Haber et al., 2016; Haber et al., 2017; Haber et al., 2019), this may change the microenvironment for resident cells. Also, the air flow near the alveolar wall might be affected by the non-uniform wall motion, which might have consequences for the deposition of particles (e.g., particulate matter, bacteria, viruses, *etc.*).

The lead author of this editorial (Akira Tsuda) became interested in the subject of this Research Topic while working on a project investigating how new alveoli are formed after pneumonectomy (Butler et al., 2012). Earlier work; e.g., Ng et al. (2005), had found that low levels of interstitial flow induce fibroblast-to-myofibroblast differentiation, collagen alignment, and fibroblast proliferation without the need for external mediators. This study suggests that the biophysical environment preceding fibrosis, involving interstitial fluid flow, may change the microenvironment within the wall, which in turn, may change the mechanical properties of the alveolar septa.

Haber et al. (2016), Haber et al. (2017) and Haber et al. (2019) showed theoretically that the nonuniform motion of alveolar walls during tidal breathing creates a unique interstitial flow and shear stress distribution in the lung’s interstitial space. Recently, Grotberg and Romanò (2023) presented a microvascular model of fluid transport in the alveolar septa, focusing on pulmonary edema. The model reveals reversed fluid flow in edema, higher than expected interstitial pressures, and suggests that the interstitium can self-clear. These finding together with the work of Ng et al. (2005) suggest that any alteration of the mechanical properties of alveolar wall (due to viral invasion, excess fluid, inhaled toxin, particulate matter, *etc.*) can generate a new interstitial flow field, and the force exerted by the interstitial flow has a major effect on the biology of the resident cells. For example, the presence of cell nuclei and fiber networks may increase local thickness and provide mechanical stability and regeneration capacity in the alveolar walls.

Another obvious cause for the alteration of septal mechanical properties is pulmonary edema. Any excess fluid in the interstitial space as well as that leaking into the air space leads to the heterogeneity of the network of alveolar wall tissue. In this Research Topic, Miserocchi nicely reviews how heterogeneity in the morphology, mechanics, and perfusion of respiratory units affects lung fluid balance. The air-blood barrier’s architecture plays a crucial role in efficient gas exchange by maintaining a thin structure and regulating extravascular water. Conditions causing edema can disrupt this balance by increasing microvascular filtration, often seen during exercise or in hypoxia-related situations. Generally, the lung can counteract increased filtration rates, but loss of control over fluid balance occurs when the macromolecular structure of lung tissue is compromised. Miserocchi suggests that such heterogeneities, which may be inherent or exacerbated by pathological processes, hinder fluid balance control and impede oxygen diffusion and transport efficiency in humans.

Furthermore, Vega et al. investigate pulmonary edema at the molecular level. They investigate the function of KvLQT1 channels and their interaction with other channels/transporters involved in ion/liquid transport using genetically modified mice and induced lung edema. Alveolar ion and fluid absorption play a vital role in maintaining lung homeostasis and resolving lung edema. Previous research has shown that KvLQT1 K+ channels contribute to the control of sodium and liquid absorption in alveolar epithelial cells. The absence of KvLQT1 channels in knockout mice resulted in a slight increase in water lung content, but lung function and structure remained largely unaffected. However, activating KvLQT1 channels reduced water lung content and enhanced the expression of other relevant transporters, suggesting the beneficial role of KvLQT1 activation in resolving lung edema.

From a global view point, Hall et al. report on a new 3D spring network model of pulmonary fibrosis, called the Amorphous Network, based on Voronoi diagrams, which more closely resembles lung geometry than past models. Pulmonary fibrosis is a fatal disease characterized by collagen deposition and lung stiffness. By introducing agents that stimulate fibroblast migration and stiffening of the network, they observe increased heterogeneity in alveolar ventilation. The model also demonstrates an increase in bulk modulus with more stiffened areas.

Finally, Liu et al. investigate cellular physiology, and find that cell size is not fixed and can respond to external factors. The researchers observed that cell surface area in the planar dimension appeared to reflect environmental changes at the organ surface layer. The liver, which experiences minimal surface changes, had large and consistent cell sizes. On the other hand, the lung, which undergoes significant surface changes during ventilation, displayed both small and large cell sizes. These changes are attributed to dynamic changes in cell shape rather than changes in cell volume. This work provides further evidence of the interaction between wall motion and cell geometry.

In summary, the natural heterogeneity of the alveolar wall network does not normally adversely affect homeostasis. However, as discussed above, many factors can upset the homeostatic balance and this imbalance can eventually lead to disease. This Research Topic highlights the interconnectedness of many of these factors, and emphasizes that a small alteration to one factor can upset the delicate balance in the alveolar wall’s ecosystem.
